# Correction: Continuous sample drop flow microextraction: a versatile and green microextraction tool in analytical chemistry

**DOI:** 10.1039/d6ra90127a

**Published:** 2026-09-21

**Authors:** Anwar Rasheed Yaqoub, Sameera Sh. Mohammed Ameen, Anwar H. Abdullah, Khalid M. Omer

**Affiliations:** a Department of Pharmacy, College of Pharmacy, Nawroz University 42001 Duhok Kurdistan Region Iraq; b Department of Chemistry, College of Science, University of Zakho 42002 Zakho Kurdistan Region Iraq; c Department of Chemistry, College of Science, University of Duhok 42001 Kurdistan Region Iraq; d Department of Chemistry, College of Science, University of Sulaimani 46002 Sulaymaniyah Kurdistan Region Iraq khalid.omer@univsul.edu.iq

## Abstract

Correction for “Continuous sample drop flow microextraction: a versatile and green microextraction tool in analytical chemistry” by Anwar Rasheed Yaqoub *et al.*, *RSC Adv.*, 2026, **16**, 29884–29904, https://doi.org/10.1039/d6ra02146h.

The authors regret an error in affiliation *c*, the affiliation for author Anwar H. Abdullah. The correct affiliation for Anwar H. Abdullah is displayed here and as follows:

Department of Chemistry, College of Science, University of Duhok, 42001, Kurdistan Region, Iraq.

The Royal Society of Chemistry apologises for these errors and any consequent inconvenience to authors and readers.

